# Multicenter Evaluation of Morbidity and Predictors of Response to Imiquimod Treatment for Penile Intraepithelial Neoplasia

**DOI:** 10.1016/j.euros.2024.08.020

**Published:** 2024-12-05

**Authors:** Ofir Avitan, Laura Elst, Manon Vreeburg, Tynisha Rafael, Katja Jordanova, Niels Graafland, Kees Hendricksen, Bas W.G. van Rhijn, Henk G. van der Poel, Maarten Albersen, Oscar Brouwer

**Affiliations:** a Department of Urology, Netherlands Cancer Institute-Antoni van Leeuwenhoek Hospital, Amsterdam, The Netherlands; b Department of Urology, University Hospitals Leuven, Leuven, Belgium; c Department of Urology, Amsterdam University Medical Centres, Amsterdam, The Netherlands

**Keywords:** Imiquimod, Penile cancer, Precancerous, Penile intraepithelial neoplasia, Complete response, Human papillomavirus

## Abstract

**Introduction and objective:**

Imiquimod (IQ) is an immunomodulator used in the management of penile intraepithelial neoplasia (PeIN) lesions. However, IQ treatment may be associated with bothersome side effects (SEs). To date, studies reporting on this morbidity and evaluating predictors of response to IQ are scarce and included small cohorts. The primary objective of our study was to assess the response to IQ treatment, associated SEs, and potential predictors of response in the largest reported cohort to date.

**Methods:**

We conducted a collaborative retrospective study involving patients diagnosed with PeIN and treated with IQ between 2010 and 2022 in two high-volume centers in the Netherlands and Belgium. Eligible patients had a confirmed diagnosis of PeIN and a minimum of 6-mo follow-up. Response to IQ was categorized as a complete response (CR), partial response, or no response. Descriptive statistics were generated and statistical tests included the Mann-Whitney U test for age and Fisher’s exact test for categorical variables.

**Key findings and limitations:**

The study included a total of 44 patients, with a median age of 65.4 yr (interquartile range 56–72). Of these patients, 28 (64%) achieved a CR, while 14 (32%) had a partial response and two (4.5%) had no response. In the CR subgroup, the 3-yr recurrence rate was 25%. No significant correlation was found between response status and age, human papillomavirus status, history of penile cancer, or circumcision before treatment. Among the patients, 50% reported SEs, mainly local pain, irritation, and bleeding, and 12% discontinued treatment because of SEs. There was no significant correlation between CR and the incidence or type of SE.

**Conclusions and clinical implications:**

Despite the high overall response rate to IQ, a significant number of patients experienced local recurrence within 3 yr, and approximately half of the patients reported SEs. Our results did not identify any clinical or pathological factors or local SEs predictive of the therapeutic response to IQ. Prospective studies are needed to help in predicting which patients are likely to respond to IQ so that those who will not benefit can be spared the SEs associated with this treatment.

**Patient summary:**

Our study looked at responses to imiquimod (IQ), an immune-based treatment in cream format, for precancerous lesions on the penis, called penile intraepithelial neoplasia. More than 95% of patients had a complete or partial response to IQ, but 50% reported side effects, and 25% of the group with a complete response had recurrence within 3 years. More research is needed to help in selecting patients who will benefit the most from IQ treatment.

## Take Home Message

The complete response rate to imiquimod treatment for penile intraepithelial neoplasia was 64% in our study, 25% of these patients experienced local recurrence and 50% of the overall cohort reported side effects. Our analyses did not reveal any significant clinical predictors of response. Prospective studies are needed to distinguish patients who are likely to respond and to mitigate imiquimod-related side effects.

## 1 Introduction

Penile intraepithelial neoplasia (PeIN) is a precancerous condition that impacts the penile epithelium and is linked to several risk factors, including lichen sclerosus. However, the most prevalent risk factor is a high-risk human papillomavirus (hrHPV) infection [1].

**Fig. 1:**
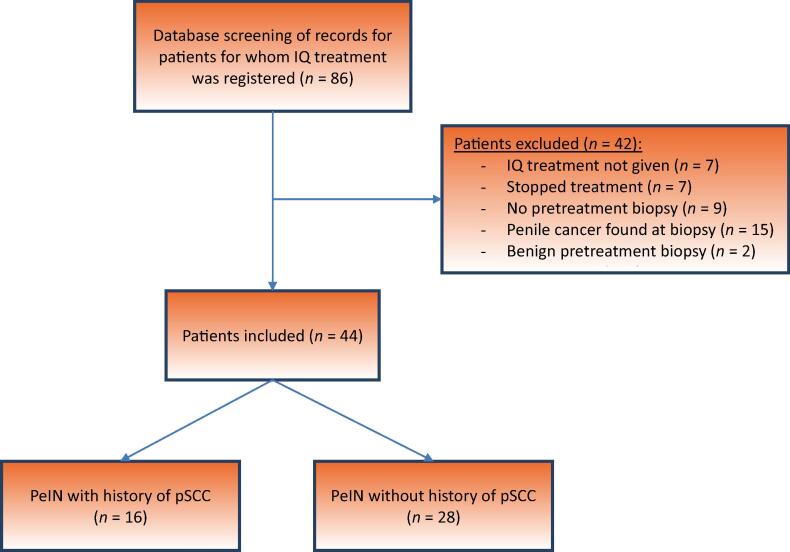
Flowchart of the database screening process with inclusion and exclusion of patients. IQ = imiquimod; PeIN = penile intraepithelial neoplasia; pSCC = penile squamous cell carcinoma.

Data on PeIN incidence rates are primarily from two retrospective studies in Swedish and Danish populations [2], [3]; although the disease remains rare, an increasing trend in recent years has been observed. The rate of PeIN progression to invasive malignancy has been reported as 5–15% [4], [5]. These findings highlight the significance of gaining a better understanding of PeIN and improving treatment strategies for patients diagnosed with this condition (Fig 1).

Current treatment options for PeIN include local intervention via excision and laser procedures, topical therapy, and a combination of topical and surgical treatments. While surgical treatments may offer lower recurrence rates than topical therapies [6], they can have a significant impact on the patient’s wellbeing. This impact may manifest through repeated treatments, disfigurement and mutilation of the penis, loss of function, and negative psychosexual consequences. Therefore, topical treatments are currently recommended as first-line therapy [7].

Imiquimod (IQ) is a well-known treatment for PeIN lesions that is widely used in clinical practice. IQ is a TLR7 agonist and acts at multiple levels of the adaptive immune system. IQ also has significant antiviral and antitumor properties, which make it suitable for both hrHPV- associated and non–hrHPV-associated lesions [8]. A recent review of studies on topical IQ treatment revealed a complete response (CR) rate of 64% and a recurrence rate of 20% [6].

The aim of our study was to investigate the side effects (SEs) and response rates to IQ treatment in a larger cohort of patients. We assessed clinical factors for potential associations with CR or disease recurrence. Such associations could motivate future research on causal mechanisms and provide guidance for clinicians in predicting patient responses to IQ treatment and managing expectations regarding treatment efficacy and recurrence.

## 2 Patients and methods

This retrospective multicenter study, carried out in collaboration between the urology departments of the Netherlands Cancer Institute in Amsterdam and University Hospital Leuven in Belgium, included patients with PeIN who were treated with 5% IQ between 2010 and 2022. Ethical approval for the study was granted by the medical ethics review board in both centers (IRBdm22-102).

Patient data including age, history of localized penile squamous cell carcinoma (pSCC), HPV status, circumcision status, treatment regimen, outcomes, SEs, and histology type were collected from medical records.

The study included patients with a confirmed histological diagnosis of PeIN and a minimum of 6-mo follow-up. Patients with a history of localized pSCC were included if they had only PeIN without concurrent pSCC. Patients without a pretreatment biopsy confirming PeIN or pSCC and those without follow-up after therapy were excluded (Fig 1).

A CR to IQ was defined as the absence of PeIN lesions on physical examination and no recurrence or negative biopsies at 6 mo after treatment. Partial response (PR) was defined as a reduction in lesion size, but with persistence or recurrence of the lesion within 6-mo follow-up and a positive biopsy after treatment. No response (NR) was defined as no change or an increase in lesion size, or histological progression. PR and NR were grouped within the PR category. HPV status was determined according to results for p16 or HPV DNA tests, with positive status assigned if either of these tests yielded positive results.

The Mann-Whitney test was used to compare patient age between the CR and PR groups. Associations between treatment response and categorical variables such as length of treatment, number of treatments per week, HPV status, and SEs were analyzed using χ^2^ tests, with Fisher’s exact test applied when the expected frequency was <5. HPV status and pretreatment circumcision, as well as SEs in relation to administration schedule and circumcision status, were examined using χ^2^ tests or Fisher’s exact test, as appropriate. The association between recurrence and treatment parameters was assessed using the same approach. Statistical analysis was performed using SPSS 26.0 (SPSS Inc., Chicago, IL, USA), with statistical significance set at *p* < 0.05.

## 3 Results

The study cohort included 44 patients who were treated with IQ. The baseline characteristics are listed in Table 1. The median patient age was of 65.4 yr (interquartile range [IQR] 56–72). A total of 64% experienced a CR to IQ treatment, and 36% had PR or NR. There were no significant differences in characteristics between the response groups. The histology reports exhibited a wide variety of terminology, with the majority of lesions not properly classified as hrHPV-related or non–hrHPV-related etiology (Table 2).

**Table 1:** Patient characteristics stratified by response to imiquimod

Parameter	Complete response	Partial/no response	*p* value
Patients (*n*)	28	16	
Median age, yr (interquartile range)	64.4 (55–73)	64.3 (57–69)	0.8
Human papillomavirus status, *n* (%)			0.4
Positive	14 (67)	7 (33)	
Negative	3 (43)	4 (57)	
Unknown	11 (69)	5 (31)	
History of localized pSCC, *n* (%)	10 (63)	6 (37)	
Circumcision, *n* (%)			0.5
Before imiquimod treatment	20 (63)	12 (37)	
After imiquimod treatment	3 (43)	4 (57)	
No	5 (100)	0	
No + after imiquimod treatment	8 (67)	4 (23)	
Side effects, *n* (%)			0.6
Yes	14 (64)	8 (36)	
No	10 (63)	6 (37)	
Unknown	4 (67)	2 (33)	

pSCC = penile squamous cell carcinoma.

**Table 2:** Histology results for PeIN lesions (*n* = 44)

Histology	Frequency, *n* (%)
**Not high-risk HPV–related**	
Differentiated PeIN	1 (2.3)
High-grade PeIN	2 (4.5)
High-grade PeIN + lichen sclerosus	1 (2.3)
Lichen sclerosus PeIN	1 (2.3)
PeIN	1 (2.3)
PeIN-III EQ	1 (2.3)
Total	7 (16)
**High-risk HPV–related**	
High-grade PeIN	4 (9.1)
HPV-related high-grade PeIN	2 (4.5)
HPV-related PeIN	1 (2.3)
HPV-related PeIN, bowenoid	1 (2.3)
HPV-related PeIN, moderate dysplasia	1 (2.3)
HPV-related PeIN, severe dysplasia	4 (9.1)
HPV-related PeIN, severe displasia, basaloid type	2 (4.5)
PeIN	3 (6.8)
PeIN, severe dysplasia, basaloid type	1 (2.3)
PeIN, severe dysplasia, bowenoid	1 (2.3)
Warty PeIN	1 (2.3)
Total	21 (48)
**HPV association not assessed**	
Differentiated high-grade PeIN	1 (2.3)
Differentiated PeIN	3 (6.8)
High-grade PeIN	7 (16)
PeIN	2 (4.5)
PeIN, moderate dysplasia	1 (2.3)
PeIN, severe dysplasia	1 (2.3)
Warty-basaloid PeIN + lichen sclerosus	1 (2.3)
Total	16 (36)

HPV = human papillomavirus; PeIN = penile intraepithelial neoplasia.

The administration schedule for local application of 5% IQ was five times a week for 6 wk in 67% of patients, and three times a week for 12 wk in 33% of them. The exact treatment schedule for two individuals was missing. Three patients received shorter treatments, one with a persistent lesion after 6 wk of treatment at three times a week treatment, and two with CR and no recurrence after 8 wk of treatment three times a week. Two patients received a longer course of treatment, one for 14 wk (3 times a week) and the other for 12 wk (5 times a week), with both showing a CR and then recurrence after 23 and 13 mo, respectively. The analysis did not reveal any significant differences in treatment response by length of treatment (*p* = 0.9) or the number of treatments per week (*p* = 0.8).

Documentation of HPV status and SEs was incomplete, with information unknown or not reported in 36% and 14% of cases, respectively. HPV status was assessed in 28 patients. The CR rate was 50% higher for HPV-positive than for HPV-negative lesions (67% vs 43%; *p* = 0.4). In the subgroup of patients who underwent circumcision before treatment and had their HPV status assessed, six were HPV-negative and 14 were HPV-positive. Notably, the group with positive HPV status had a higher CR rate (71%) than the group with negative HPV status (33%). However, the difference was not statistically significant (*p* = 0.2).

SE data were available for 38 patients (86%), of whom 16 (42%) reported no SE. The most common SEs were pain and irritation, each reported by 32% of patients, followed by local bleeding (18%). There was no significant correlation between the SE rate or type and CR or PR (Table 3).

**Table 3:** Side effects as reported in patient records (*n* = 38)

Side effect	Patients, *n* (%)			*p* value
	Complete response	Partial/no response	Overall cohort	
Pain	7 (29)	5 (36)	12 (32)	0.7
Irritation	7 (29)	5 (36)	12 (32)	0.7
Erythema	0 (0.0)	2 (14)	2 (5.3)	0.1
Bleeding	6 (25)	1 (8.0)	7 (18)	0.2
Ulcer	1 (4.2)	1 (7.0)	2 (5.1)	0.6
Any side effect	14 (58)	8 (57)	22 (58)	0.6

Some 12% of patients, comprising 6/7 cases excluded for discontinuation of IQ treatment, discontinued their treatment prematurely because of SEs. There was no significant correlation between SEs and the IQ administration schedule (*p* = 0.7). Despite the lack of statistical significance for a correlation between SEs and circumcision status, a lower SE rate was observed for patients who underwent circumcision before treatment (52%) than for those who were circumcised after treatment or not at all (86%; *p* = 0.2).

Among the 28 patients with a CR, mean follow-up was 19.8 mo (range 5.7–142.8). Seven patients experienced recurrence, with a median time to recurrence of 14.2 mo (IQR 12–23). No significant correlations were found between recurrence and the treatment schedule, number of treatments, or treatment length.

## 4 Discussion

We evaluated IQ treatment for PeIN in terms of SEs, response, and potential predictors of response in a relatively large cohort of 44 patients from two reference centers. Our findings showed a relatively high response rate to IQ treatment, with a CR in approximately two-thirds of patients. We tested the association of several clinical variables with IQ response status, but no significant correlations were observed.

Several small studies have assessed the CR rate for IQ treatment in PeIN, considering factors such as a history of penile cancer or PeIN, previous therapies, lesion characteristics, HPV status, smoking habit, immunocompromised state, and treatment schedule. A 2017 review [9] of 48 patients from 29 studies reported an overall CR rate of 63%, but potential selection bias because of mostly single case reports should be noted. In a recent retrospective eUROGEN study [4], a CR rate of 100% was observed for a cohort of 16 PeIN patients treated with different therapy sequences involving IQ. This illustrates the potential of IQ use for first- or second-line treatment of PeIN. Studies on vulvar intraepithelial neoplasia (VIN) can be used as a point of reference for the CR rate. Randomized controlled trials (RCTs) in VIN reported CR rates of ∼80% [10], [11], while a different RCT [12] had a lower rate of 35%. Systematic reviews confirmed a wide CR rate for VIN patients of 5–100% [13], [14]. The variations in CR rates may be attributable to differences in lesions, treatment approaches, and the size of the study populations. We observed a CR rate of 64%, which aligns with the average rate found in those studies. Nevertheless, there is notable variability in response rates across studies, underscoring the significance of the use of clinical and histological factors to predict treatment response.

Our study did not reveal any clinical parameters that were significantly associated with the response to IQ. Removal of the prepuce did not notably enhance the CR rate in the HPV-negative group, contrary to expectations. A study investigating pretreatment circumcision reported a CR rate of 74% in comparison to 50% with local 5-fluorouracil alone [15]. The predictive significance of HPV status has been identified as a favorable marker in penile cancer [16], [17], [18]; however, its predictive significance for response to IQ treatment in PeIN lesions remains uncertain. A study by Westermann et al [19] addressed this issue and found that CR rates were higher (84% v. 65%) and recurrence rates were lower (11% vs 38%) for HPV-positive versus HPV-negative VIN lesions. Our results revealed no statistically significant difference in CR or recurrence rates between the HPV-positive and HPV-negative groups. However, the CR rates was approximately 50% higher for the HPV-positive group. Nonetheless, HPV negativity should not be the sole factor in decisions to exclude patients from IQ treatment, as HPV-negative cases still showed notable response rates. HPV status may be considered when determining the treatment schedule, as cutaneous malignancies often require treatment at higher doses or frequencies when there is no or only mild local reaction during treatment [20]. Notably, only 74% of our cohort had documented HPV status, and the number of HPV-negative lesions was relatively small. Thus, further research is needed to confirm our findings. If no clinical predictors are discovered, another avenue to explore could be translational research in term of immune markers, gene methylation, and molecular or genetic predictors [6], [18].

Recurrence rates are vital for assessing treatment effectiveness and determining the need for further follow-up or therapy. A recent review found a recurrence rate of 20% among 25 patients who received IQ treatment [6]. Among studies on IQ treatment for VIN lesions the average recurrence rate was 33%, ranging from 0 to 100% [14]. In another retrospective study involving 20 VIN patients the recurrence rate was 35%, with a median time to recurrence of 19.7 mo (range 3.2–32.7) [21]. In our study the 3-yr recurrence rate was 25% in the CR group, falling between the average rates reported from PeIN studies and VIN studies. The recurrence rate varies among studies and depends on the definition and duration of follow-up. In addition, the longest time to recurrence was nearly 3 yr, with an average of 17.7 mo. This highlights the importance of extended follow-up beyond 3 yr for CR cases, especially in patients not capable of self-examination. Overall, the recurrence rates reported vary because of differences in study methodology, definitions, and follow-up duration. When interpreting these findings, it is crucial to consider the range of recurrence rates from various studies. We found no correlation between clinical variables and recurrence. Larger studies with defined follow-up periods are necessary for more accurate conclusions regarding the relationship between clinical variables and PeIN recurrence.

The literature on PeIN does not address the ability of SEs to predict the response to IQ treatment. However, insights into the correlation between SEs and treatment response can be gained from studies on skin lesions, as IQ is used to treat basal cell carcinoma (BCC) and precancerous SCC lesions. An RCT systematically assessing the local reaction in patients with BCC treated with IQ as absent, mild, moderate, or severe revealed correlation between higher local reaction severity and a higher BCC clearance rate [22]. These results offer valuable insight into the potential correlation between SEs and response to IQ treatment in PeIN, although further research is required to validate the findings. We found no correlation between SEs and response rates. SE registration and follow-up for our patients were primarily recorded in a subjective manner, and the local reaction was not evaluated. Hence, future studies should investigate any correlation between SEs and response using a well-described method.

According to the World Health Organization 2022 classification [23], PeIN should be divided into two groups according to HPV status. All HPV-related lesions, regardless of the degree of cytoarchitectural features within the lesion, should be classified as high-grade lesions and referred to as HPV-related HG PeIN, while non–HPV-related lesions should be classified as differentiated PeIN. Our study data included highly variable terminology (Table 2), and many histology reports lacked information on HPV status. Categorization according to HPV status could enhance the comparability of results in future studies and assist in predicting prognosis and response to various treatments, especially for IQ.

The retrospective nature of our study means that it has certain inherent limitations that must be acknowledged. The other main limitation is the small sample size, which limits the strength of our conclusions. For instance, 28/44 patients has a CR, but only seven of these patients were HPV-negative. A statistically significant difference in response rates between HPV status groups would be observed only if one HPV-negative patient at most had a CR. If the true response rates were, for example, 80% for the HPV-positive group and 40% for the HPV-negative group (a difference we would consider clinically significant), the power for detection of this difference would only be approximately 15%. This power analysis indicates that given our sample size and the imbalance between the CR and PR groups, the study lacks power to detect small to moderate effect sizes. Therefore, the absence of statistically significant differences in clinical parameters does not conclusively rule out their potential predictive value. Future studies with larger sample sizes are necessary to confirm our observations and identify potential predictors of treatment response.

Despite the lack of significant associations in our results, prospective studies should examine potential factors predictive of better response rates and recurrence-free outcomes. These factors should include pretreatment circumcision, which enhances the ease of application and exposure to local IQ treatment; HPV status, which might influence the treatment response; and the treatment schedule, administration frequency, and dosage, which could impact outcomes, as local side effects may necessitate adjustments.

This study is underpowered and did not find significant associations. Investigation of these factors in prospective studies, including assessment of HPV status for all patients, thorough follow-up, and accurate recording treatment schedules and any necessary adjustments, could identify significant results for inclusion in multivariable analyses to determine their interrelations and potential as predictors of treatment efficacy.

## 5 Conclusions

Despite the high overall response rate to IQ, a significant number of patients with PeIN experienced local recurrences within 3 yr, and approximately half of the patients reported side effects. Clinical or pathological factors and local side effects did not predict the therapeutic response to IQ in the current study. Prospective studies are needed to help in predicting which patients are likely to respond to IQ so that those who are not likely to benefit can be spared the side effects associated with IQ.

  ***Author contributions:*** Ofir Avitan had full access to all the data in the study and takes responsibility for the integrity of the data and the accuracy of the data analysis.

  *Study concept and design*: Brouwer.

*Acquisition of data*: Avitan, Elst.

*Analysis and interpretation of data*: Avitan.

*Drafting of the manuscript*: Avitan.

*Critical revision of the manuscript for important intellectual content*: Rafael, Vreeburg, Elst, Brouwer.

*Statistical analysis*: Avitan.

*Obtaining funding*: None.

*Administrative, technical, or material support*: Jordanova, Graafland, Hendricksen, van Rhijn, van der Poel.

*Supervision*: Albersen, Brouwer.

*Other*: None.

  ***Financial disclosures:*** Ofir Avitan certifies that all conflicts of interest, including specific financial interests and relationships and affiliations relevant to the subject matter or materials discussed in the manuscript (eg, employment/affiliation, grants or funding, consultancies, honoraria, stock ownership or options, expert testimony, royalties, or patents filed, received, or pending), are the following: None.

  ***Funding/Support and role of the sponsor:*** None.
